# Development and Beta Validation of an mHealth-Based Hearing Screener (SRESHT) for Young Children in Resource-Limited Countries: Pilot Validation Study

**DOI:** 10.2196/53460

**Published:** 2025-01-13

**Authors:** Vidya Ramkumar, Deepashree Joshi B, Anil Prabhakar, James W Hall, Ramya Vaidyanath

**Affiliations:** 1 Faculty of Audiology and Speech Language Pathology Sri Ramachandra Institute of Higher Education and Research Chennai India; 2 Department of Electrical Engineering Indian Institute of Technology Madras Chennai India; 3 Osborne College of Audiology Salus University Pennsylvania, PA United States

**Keywords:** audiometry, mHealth, devices, wireless, tablet-based screening, childhood hearing loss, early hearing detection and intervention, tablets, children, neonates, hearing loss, infants, development, validation, mobile phones

## Abstract

**Background:**

The prevalence of hearing loss in infants in India varies between 4 and 5 per 1000. Objective-based otoacoustic emissions and auditory brainstem response have been used in high-income countries for establishing early hearing screening and intervention programs. Nevertheless, the use of objective screening tests in low- and middle-income countries (LMICs) such as India is not feasible. Mobile health (mHealth) solutions have been demonstrated to be a viable option for hearing screening in LMICs.

**Objective:**

This study aims to develop and beta-validate an affordable hearing screener for children younger than 6 years of age to identify moderately severe or higher degrees of hearing loss.

**Methods:**

In phase 1, a mHealth-based hearing screener (SRESHT) was developed using a single board computer with wireless commercial headphones and speakers as transducers, which were calibrated according to the standard procedure. Three subjective hearing screening modules were conceptualized and developed for different age groups: (1) behavioral observation audiometry–screening for infants aged from 0 to 1 year; (2) speech spectrum awareness task–screening for children 1 to 3 years old; and (3) speech recognition task–screening for children 3 to 6 years old. Different auditory stimuli for the screening modules were generated and suitability was assessed: (1) noisemakers, animal sounds, and environmental sounds for infants (birth to 1 year old); (2) animal sounds and nonsense syllables for children (1 to 3 years old); and (3) eighteen picturable spondee words for children (3 to 6 years old). In phase 2, the SRESHT screener was beta-validated in children aged below 6 years to establish the agreement between SRESHT modules and the gold-standard procedure in identifying moderately severe and higher degrees of hearing loss.

**Results:**

Off-the-shelf commercial speakers and headphones were selected and calibrated. On comparison of stimuli for behavioral observation audiometry on 15 children, Noisemaker stimuli were found suitable based on the average minimum response levels. On comparison of different stimuli for speech spectrum awareness task on 15 children, animal sounds were found to be suitable. On familiarity check of 18 spondee words for speech recognition task among 20 children, 12 spondee words had the eligibility cutoff (85%) and a presentation level of 5 dB SL (re-pure tone threshold) was sufficient to achieve 80% psychometric function. In phase 2, a total of 55 children aged 0 to 6 years (31 normal hearing and 24 hearing impairment) underwent SRESHT screening for beta validation. Cohen κ indicated that the overall SRESHT screener had a very good agreement (κ=0.82) with gold-standard audiometric screening for identifying moderately severe and higher degrees of hearing loss.

**Conclusions:**

The development and beta validation of the SRESHT screener using the selected auditory stimuli showed that the stimuli were suitable for screening children.

## Introduction

Childhood hearing loss can have wide-ranging effects on a child’s language, speech, education, and social integration, making this a crucial issue to address. To achieve optimal outcomes using amplification devices, it is necessary to screen, identify, and intervene children before 6 months of age [1]. In high-income countries, there are often policies for newborn hearing screening and hearing screening of young school-aged children [1]. On the other hand, in low- and middle-income countries (LMICs), such mandatory policies do not exist.

In India, the prevalence of hearing loss among neonates and infants is 5 per 1000 [2], and it ranges between 6% and 16% among children [3]. However, procuring and maintaining the recommended objective screening instruments like otoacoustic emissions and auditory brainstem response has not been viable at scale. Even in several other LMICs, only paper-pencil–based checklists are used to screen hearing loss, for example, Bangladesh [4], Thailand [5], Ecuador [6], Kenya [7], and Brazil [8]. The problem is further magnified by the lack of adequate ear and hearing care providers for early identification and rehabilitation of children with hearing loss. For example, in India, the ratio of audiologists to the population as per recent data from the Rehabilitation Council of India is 4.41 per 100,000 population [9] and in Bangladesh, the ratio of audiometrists to the population is 0.77% [10].

Task shifting to community workers or health care volunteers is a strategy that has been recommended [11] and explored [12-14] to overcome the stark service gaps. Such task shifting has resulted in successful implementation by improving program outcomes like coverage, refer rate, and follow-up rate [12,14]. This has been well supported by innovations in mobile health (mHealth). mHealth apps have decentralized hearing care from tertiary and secondary care hospitals to primary and community settings in some of the LMICs [14-16]. However, many of the mHealth solutions are focused on adult hearing loss and hearing aid fitting [17-20]. Smartphone or tablet-based apps for children are only available for those over the age of 4 years [21-24] and use either pure tones [22], digit in noise [25], or speech stimuli [21]. Therefore, there is a need for an affordable mHealth screener for younger children that can be used by community workers to accurately screen hearing loss and support early intervention in LMICs.

In general, subjective measures are less preferred for screening due to their lower reliability. For example, behavioral observation audiometry (BOA) is reported to be less accurate as it is often performed informally using uncalibrated stimuli without standardization of intensity, frequency, and distance [11,26-28]. Yet, recognizing the inequity in the availability of testing tools, the World Health Organization’s guidelines for implementing community-based childhood hearing screening include the recommendation of using subjective-based screening tools [29].

Therefore, this study describes the development of a tablet-based subjective hearing screener (SRESHT screener) to identify children younger than 6 years of age with moderately severe hearing loss or higher degrees of hearing loss as per the definition of the Global Experts Group [30]. Since the variability of subjective measures is higher only for lower degrees of hearing loss [31,32], measures such as BOA and speech awareness and speech recognition–based screening modules were considered affordable alternatives to screen for hearing loss (HL; 60 dB and greater in the better ear). This preliminary level (60 dB HL and greater) of screening was chosen as part of a larger feasibility study to screen for hearing losses that are eligible for publicly funded welfare schemes in India. Similarly, in many other LMICs (eg, Bangladesh, Brazil), moderate or higher degrees of hearing loss are considered for welfare schemes [33], aids, and appliances with public funds. Therefore, the screening device is designed for application in similar contexts.

The specific objectives were to (1) identify the most appropriate auditory stimuli to screen for hearing loss in children aged between 0 and 6 years and (2) to evaluate the agreement between the pass or refer results of from behavioral screening using gold standard audiometer and of screening device in screening for moderately severe and above degrees of hearing loss.

## Methods

### Ethical Considerations

This cross-sectional study was approved by the institutional ethics committee of Sri Ramachandra Institute of Higher Education and Research (IEC-NI/20/OCT/76/113). Informed consent was obtained from parents of children who participated in the study. The subjects were included on a voluntary basis and no monetary benefits were given to the participants. All subjects were anonymized during data entry, analysis, and reporting to maintain confidentiality.

### Development of SRESHT Hearing Screener

#### Hardware and Software

A single board computer was used as the hardware with software coded using Python (Python Software Foundation) language. App modules were developed for hearing screening for children from birth to 6 years of age. The user interface was designed in both English and the local language with simple texts, images, and symbols such that nontechnical screening personnel can use the software. Three hearing screening test procedures were included: BOA-based screening (for children younger than 1 year), speech spectrum awareness task (SSAT)–based screening (for children older than 1 year and younger than 3 years), and speech recognition task (SRT)–based screening (for children older than 3 years and younger than 6 years).

The test module gets automatically selected based on the age selection in the app by the tester. The test modules include screening frequencies of 500 Hz, 1000 Hz, 2000 Hz, and 4000 Hz at a specified moderate intensity level (selected based on calibration). For each test procedure, age-appropriate stimuli were developed (described under each test procedure in detail) and uploaded to the software. An inbuilt noise monitoring software was included which provides access to the screening module only when the ambient noise levels are less than 50 dB (A). A cloud-based back-end data management was integrated via Google Drive. The screening result is displayed automatically based on the response entered for each frequency.

#### Selection of Transducers

Suitable commercial transducers appropriate to the test module and age were selected based on frequency response at 500 Hz to 4 kHz, output intensity range, and distortions at intensities up to 80 dB SPL (sound pressure level). At first, 3 commercial grade supra-aural headphones (Sony WHCH510 headphones, Sennheiser HD 450 BT, and Boatrockers 410), and 3 speakers (JBL Go 2, Sony SRSXB12, and Fire Boltt Xplode 1500) were selected for output comparison based on manufacturer information on frequency, intensity range, cost, and ease of availability in the market.

#### Headphone

Headphones were connected via Bluetooth to the SRESHT screener at maximum volume setting. The headphone diaphragm was coupled to the calibration microphone with a 6CC coupler connected to the Larsen Davis sound level meter (SLM).

#### Speaker

Bluetooth speakers were connected to the SRESHT screener at maximum volume setting and placed at a distance of 1 m and at 0-degree azimuth from the SLM microphone. A subject was seated in a chair and the ear level was marked from the floor. The subject was then removed and the SLM microphone was suspended from a stand approximately at the ear level marked.

For all the transducers, warble tone stimuli were presented at 500 Hz, 1000 Hz, 2000 Hz, and 4000 Hz at different volume settings (%) of the SRESHT screener hardware, and the corresponding dB SPL values were measured in the SLM. The software was initially coded to produce the desired output level for moderate intensity levels in SPL (40 dB, 50 dB, and 60 dB) across the frequencies.

The Sony WHCH510 headphones and JBL Go 2 pro speakers were selected based on their accuracy of output, consistency of output, and output range (described in Development of the SRESHT Screener section).

#### Reference Equivalent Threshold Sound Pressure Level of Transducers

##### Sony WHCH510 Headphones

The mean thresholds of 10 normal hearing individuals were measured on a standard audiometer using TDH39 headphones from 500 Hz to 4000 Hz. The same individuals’ threshold volume levels (%) were then obtained using the SRESHT hearing screener and Sony WHCH510 headphones. Larson Davis SLM was used to calculate the dB SPL value corresponding to the threshold volume level for each frequency. The Wilber method was used to estimate the reference equivalent threshold sound pressure level (RETSPL) values for the Sony WHCH510 headphones [34].

##### JBL Go2 RETSPL

The standard RETSPL values for speakers at 0-degree azimuth [35] were applied for 500 Hz to 4 kHz. The software was recoded as per the RETSPL value derivation.

### Calibration Tone

A 1000 Hz warble tone was generated and used as a calibration tone, which is introduced in the app prior to the commencement of the screening. The minimum audible level of the tone at a moderate noisy environment of 40 to 45 dBA for 10 normal hearing subjects was measured and used to set the calibration tone.

### Stimulus Validation

#### BOA-Based Hearing Screening

Both tonal and nontonal stimuli were assessed for BOA [32,36]. Studies suggested that children’s responses to nontonal stimuli were as effective as tonal stimuli [36]. Nontonal stimuli included environmental sounds, animal sounds, and noisemakers that were contextually relevant to screen children. The stimuli were downloaded from an open-source website [37,38]. The acoustic parameters of the stimuli were then modified and corrected or normalized using Audacity (version 3.5.0; Audacity Team) and Apple Logic Pro (version 10.6; Apple Inc). The stimuli and the characteristics are summarized in Table 1.

**Table 1:** Nontonal stimuli categorized into noisemakers, environmental sounds, and animal sounds with center frequencies of 500 Hz, 1000 Hz, 2000 Hz, and 4000 Hz and a bandpass filter with 500-Hz bandwidth (filter slope 24 dB/octave).

Frequencies (center frequency, Hz)	Noisemakers	Environmental sounds	Animal sounds	Bandwidth required	Duration (seconds)
500	Drum or wooden drum	Horn	Cow mooing	250 Hz to 750 Hz (18 or 24 dB/octave slope)	2-3
1000	Wooden rattles	Applause	Dog barking	750 Hz to 1250 Hz (24 dB/octave slope)	2-3
2000	Metallic rattle or squeaking toy	Laughing	Whinnying horse and goat meh	1750 to 2250 Hz (24 dB/octave slope)	2-3
4000	Bell	Telephone ringing	Bird chirp and cat meow	3750 to 4250 Hz (24 dB/octave slope)	2-3

#### Procedure for Identifying Suitable Stimuli for BOA

A sound-treated room was preferred for conducting the screening test at the SRIHER clinic or laboratory. Participants were children with normal hearing and hearing loss between 0 to 1 year of age. Participants’ details are described in Table 2. A standard diagnostic audiometer (Madsen Astera^2^) with calibrated speakers (Martin Audio C115) was used to conduct the test using the newly developed stimuli (warble, noisemakers, environmental sounds, and animal sounds) at 500 Hz, 1000 Hz, 2000 Hz, and 4000 Hz for children. Behavioral responses such as eyeblink, eye widening, searching, and head turn toward the source were considered acceptable responses. This was noted by an audiology intern. Initially, the stimuli presentation started at 20 dB HL in 500 Hz for children with normal hearing and at a higher intensity level for children with hearing loss. If a response was obtained, the stimuli presentation was presented thrice in the same intensity level to confirm the response and if not, the presentation was increased by 5 dB steps (ascending method). Whenever the child got distracted or bored, tangible or visual reinforcement was given by the audiology assistant. The same procedure was repeated for each stimulus at each frequency. Minimum response levels or thresholds were obtained for each stimulus at each frequency and compared to identify the stimuli that resulted in the lowest thresholds.

**Table 2:** Characteristics of children involved in BOA^a^ stimuli validation.

Participants	Sex	Age	Hearing age	Diagnosis	Type of amplification
Child 1	Male	6 months	6 months	Normal hearing	N/A^b^
Child 2	Female	4 months	4 months	Normal hearing	N/A
Child 3	Male	6 months	6 months	Normal hearing	N/A
Child 4	Male	6 months	6 months	Normal hearing	N/A
Child 5	Male	6 months	6 months	Normal hearing	N/A
Child 6	Female	4 months	4 months	Normal hearing	N/A
Child 7	Female	8 months	8 months	Normal hearing	N/A
Child 8	Male	8 months	8 months	Normal hearing	N/A
Child 9	Male	3 months	3 months	Normal hearing	N/A
Child 10	Female	9 months	9 months	Normal hearing	N/A
Child 11	Female	5 months	5 months	Normal hearing	N/A
Child 12	Male	2 years 5 months	8 months	Bilateral: Severe to profound hearing loss	Right: Cochlear implant
Child 13	Male	2 years 6 months	10 months	Bilateral: Severe to profound hearing loss	Right: Cochlear implant
Child 14	Female	1 year 8 months	6 months	Bilateral: Moderately severe hearing loss	Bilateral: Hearing aid
Child 15	Female	2 years 2 months	8 months	Bilateral: Moderately severe hearing loss	Bilateral: Hearing aid

^a^BOA: behavioral observation audiometry.

^b^N/A: not applicable.

#### SSAT-Based Hearing Screening

For children between 1 and 3 years of age, speech-like animal sounds, such as a cows’ *moo*, dogs’ *bow-wow*, goats’ *meh*, and cats’ *meow,* and nonsense syllables like *mamama* and *bababa* were included*.*

The spectrum of the animal sounds using PRAAT software (version 6.3.2; Softsonic International) was compared to the spectrum of consonants and vowels to verify that it resembled the properties of speech. The formant frequencies (F1, F2, and F3) of animal sounds were approximately similar to the corresponding formant frequencies of the target vowels. Table 3 shows the formant frequencies of recorded animal sounds and existing normative vowel formant frequencies, as adapted from Raphael et al [39].

**Table 3:** Comparison of formant frequencies of animal sounds and normative formant frequencies of vowels matched approximately^a^.

Formant frequencies	Animal sounds			
	Cow moo/ /u/	Dog bark/ /a/	Goat meh/ /ae/	Cat meow/ /i/
F1	300 Hz/300 Hz	853 Hz/ 730-850 Hz	1565 Hz/ 850-1100 Hz	1566 Hz/ 1500 Hz
F2	693 Hz/800 Hz	1180 Hz/1100-1220 Hz	2060 Hz/2050-2300 Hz	2217 Hz/2500 Hz
F3	986 Hz/2100 Hz	1616 Hz/2100-2400 Hz	2507 Hz/ 2800-3200 Hz	2835 Hz/ 3000 Hz

^a^/u/,/a/,/ae/,/i/ are English vowel sounds represented in international phonetic alphabet (IPA).

#### Procedure for Identifying Suitable Stimuli for SSAT

For children between 1 and 3 years of age, SSAT-based screening was conducted with the standard audiometer (Madsen Astera^2^), with TDH 39 headphones with newly developed stimuli. The participants included children with normal hearing and hearing impairment between the ages of 1 and 3 years. Behavioral responses included head turn, locating the source of the sound, and imitating the sound. Stimuli was presented initially at 1 kHz at 60 dB HL for normal hearing and 80 dB HL for children with hearing impairment. The Hughson-Westlake method of threshold tracking was used to identify the minimum response levels for each stimulus at each frequency. The minimum response levels for these stimuli were compared and the stimuli yielding better responses (thresholds) were finalized for the screening test procedure.

#### SRT-Based Hearing Screening

SRT-based screening was developed for children between 3 and 6 years of age. Eighteen picturable spondee words in the local language (Tamil) were excerpted from a standardized spondee list of 50 words [40] that is routinely used in clinical practice. The words were selected based on the relevance of the word in present-day context, and its picturability. Pictures corresponding to the 18 words were downloaded from the open-source web-based repository named Wikimedia Commons [41].

#### Procedure for Identifying Suitable Stimuli for SRT

A familiarity test of all the words and their corresponding picture was conducted. Participants were both children with normal hearing and children with hearing impairment above the age of 3 years. For familiarity testing of words, each child was asked to point out the appropriate picture when the word was read out at the conversation level. Similarly, for picture familiarity, each child was asked to name the picture.

These 18 spondee words were further divided into 3 lists of 6 words each. Then, these spondee words were recorded with a female voice centered at 1000 Hz and with loudness balanced at –3 dB peak value, having an interstimulus interval of 5 seconds. A closed-choice response set was developed with 1 target picture and 3 other pictures. The 4 pictures were arranged in a sequential manner with an equal number of repetitions.

#### Procedure for Determining Suitable Presentation Level for SRT Screening in Children

Participants were children with normal hearing and children with hearing impairment between the age group of 3 and 9 years. Using the standard audiometer (Madsen Astera^2^) with TDH 39 headphones, each child’s pure tone thresholds (PTA) were obtained using the Hughson-Westlake method. Then, each child underwent SRT with picture identification as response. This was performed at different sensation levels (0 dB SL, 5 dB SL, 10 dB SL, and 20 dB SL with re-PTA) to identify the level at which 80% correct response (5/6 words) was obtained.

### Beta Validation of SRESHT Screening Modules

This beta validation was conducted prior to the full validation in which the suitability of the stimuli was evaluated and agreement between new stimuli versus standard stimuli was compared.

#### Sample

Two groups of children participated in the study. The first group consisted of children under 6 years of age with normal hearing. The other group consisted of children with hearing impairment having a hearing age of less than 6 years and these children were younger than 10 years of chronological age. Children with any other comorbid conditions or any other known disabilities were excluded from the study (eg, children with developmental delay, vision impairment, autism spectrum disorders).

Children were recruited from the outpatient clinic of the tertiary care hospital attached to the institution where the study was conducted, as well as in camps organized in 2 rural districts of Tamil Nadu for the period of 3 months.

Informed written consent was obtained from the parents of the children before the commencement of data collection.

#### Procedure

The procedure was conducted in any quiet room near the outpatient department or the campsites with an ambient noise level of less than 50 dB (A) as recorded through the noise monitoring software of the SRESHT screener. SRESHT hearing screening with the finalized stimuli and screening modules for each age group was performed for all children using age-appropriate screening procedures at 60 dB HL.

Then, the minimum response level was estimated for 500 Hz, 1 kHz, 2 kHz, and 4 kHz with the standard diagnostic audiometer (Madsen Astera^2^) for each test procedure, as a gold standard. Screening results for the SRESHT hearing screener at 60 dB HL (pass or refer) were compared to audiometric thresholds more or less than 60 dB HL.

#### Analysis

Nonparametric tests were used to analyze the data as the results of the Shapiro-Wilk test showed that the data were not normally distributed.

The Friedman test was used to compare the children’s responses to different stimuli in BOA. The Wilcoxon signed rank test was used to compare the children’s responses to different stimuli for SSAT. Percentage analysis was used for word familiarity for SRT screening. Agreement statistics were used to beta-validate the screening results.

## Results

### Development of SRESHT Hearing Screener

#### Transducers

The summary of output characteristics of various speakers and headphones is shown in Table 4. The Sony WHCH510 headphones and JBL Go 2 pro speakers were selected based on their accuracy of output, consistency of output, output range, ease of use, and cost.

**Table 4:** Characteristics of each transducer (speakers or headphones) for the hearing screening module.

Transducers	Frequency response	Minimum output	Maximum output	Distortion
Sony WHCH510 headphones	No frequency splattering from 500 Hz to 4 kHz	40 dB SPL	100 dB SPL	Within permissible limits (ANSI^a^, 2020)
Sennheiser HD450BT headphones	No frequency splattering from 500 Hz to 4 kHz	40 dB SPL	102 dB SPL	Within permissible limits (ANSI, 2020)
Boat rockers 410 headphones	Frequency splattering present at 4 kHz	50 dB SPL	104 dB SPL	Distortion present at 2 kHz and 4 kHz at 80 dB SPL
JBL Go 2 speakers	No frequency splattering from 500 Hz to 4 kHz	40 dB SPL	80 dB SPL	Within permissible limits (ANSI, 2020)
Sony SRSXB12 speakers	Appropriate from 500 Hz to 4 kHz	40 dB SPL	77 dB SPL	Within permissible limits (ANSI, 2020)
Fire Boltt Xplode1500 speakers	Frequency splattering present 2 kHz and 4 kHz	50 dB SPL	87 dB SPL	Distortion presented 2 kHz and 4 kHz at 80 dB SPL

^a^ANSI: American National Standards Institute [42].

#### RETSPL of Transducers

The RETSPL values derived for the Sony WHCH510 headphones are shown in Table 5.

**Table 5:** Sony WHCH510 headphone RETSPL^a^ values.

Frequency	RETSPL (dB)
500 Hz	11
1 kHz	1
2 kHz	3
4 kHz	–2

^a^RETSPL: reference equivalent threshold sound pressure level.

### Stimuli Validation

#### BOA-Based Hearing Screening

Fifteen children (11 Children with normal hearing and 4 Children with hearing impairment) in the age range of 0 to 1 year of age with a mean age of 6 (SD 1.9) months for children with normal hearing and a mean hearing age of 8 (SD 1.6) months for children with hearing impairment (Table 2), underwent the BOA using the newly developed stimuli. The Friedman test revealed no significant difference in the average minimum response levels of warble tone versus average minimum response levels of noisemakers, animal sounds, and environmental sounds (n=15; *χ*^2^_3_=15.44; *P*=.089).

**Figure 1:**
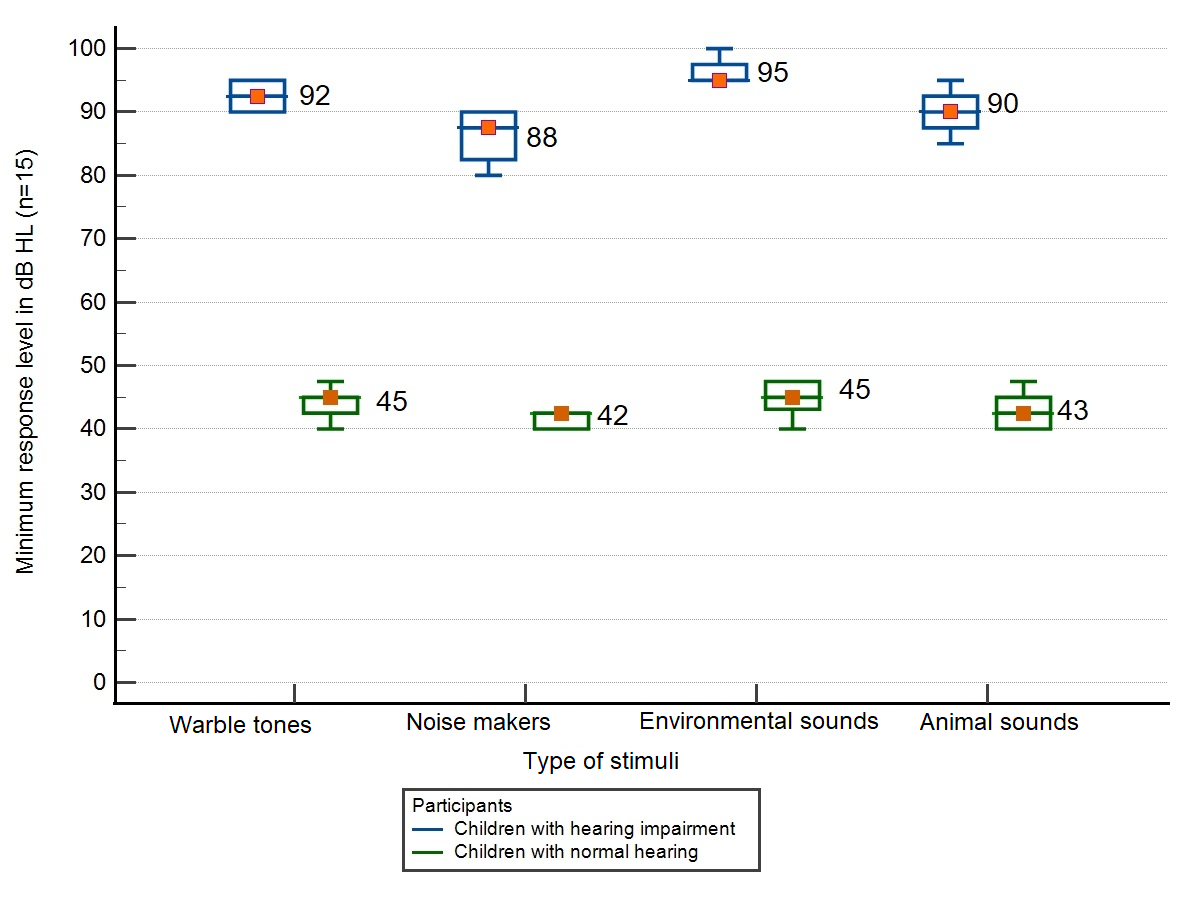
Comparison of average minimum response levels for tonal and nontonal stimuli among children with normal hearing and children with hearing loss.

A comparison of the average minimum response levels for different stimuli for children with normal hearing and children with hearing impairment revealed that the average minimum responses to noisemakers for children with hearing impairment were superior to the responses to other stimuli, that is, animal sounds and environmental sounds (Figure 1). Therefore, noisemaker-based stimuli were selected for this age group, even though all 3 categories of non-tonal stimuli were appropriate for hearing screening.

##### SSAT-Based Screening

**Figure 2:**
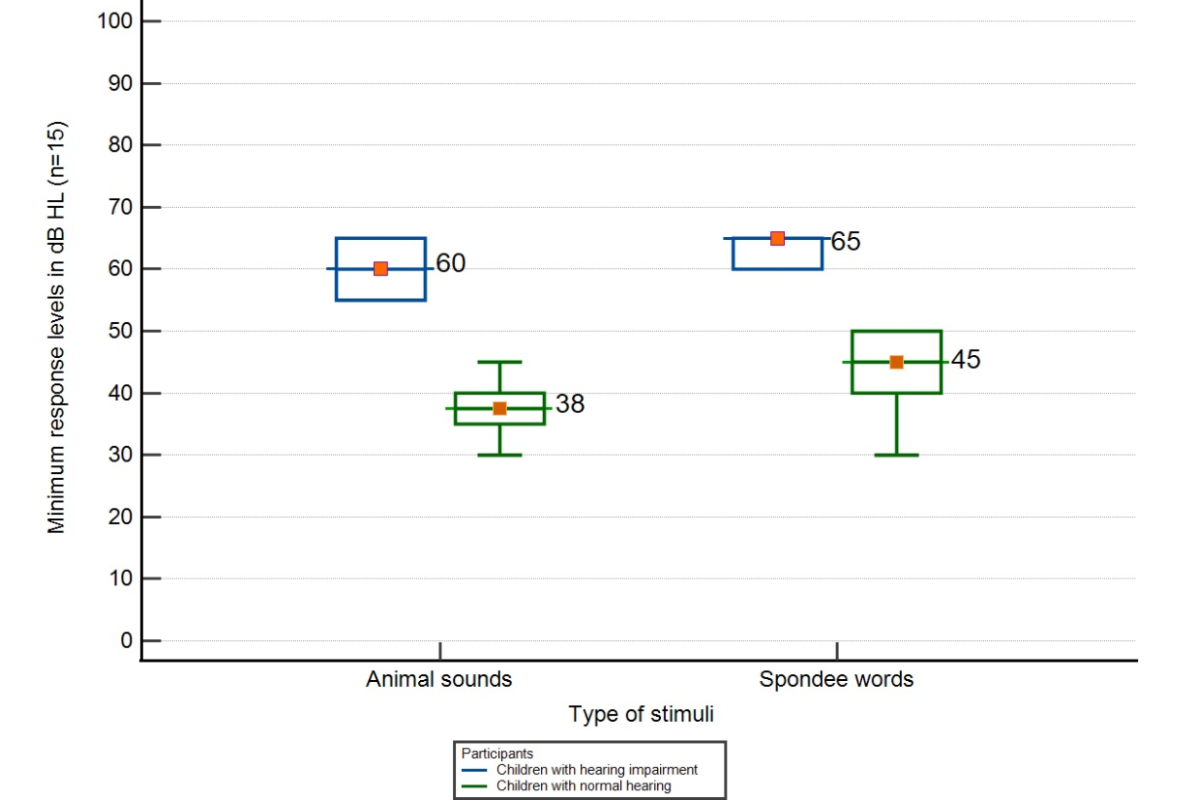
Comparison of average minimum response levels for different stimuli among children with normal hearing and hearing loss (HL).

A total of 15 children (10 children with normal hearing and 5 children with hearing impairment) in the age range of 1 to 3 years with a mean age of 2.3 (SD 0.37) years for children with normal hearing and 1.7 (SD 0.26) years of hearing age for children with hearing impairment (Table 6) underwent the SSAT. Using animal sound stimuli centered as frequencies corresponding to different speech sounds. Wilcoxon signed rank test showed a significant difference in the threshold for the nonsense syllables response and animal sounds response (*z*=–3.59; *P*<.001). The mean minimum response level difference between animal sounds was 5 dB better than the mean minimum response levels of nonsense syllables (Figure 2). Therefore, animal sounds were finalized for this age group.

**Table 6:** Participants characteristics of children involved in SSAT^a^ stimuli validation.

Participants	Sex	Chronological age (in years)	Hearing age (in years)	Diagnosis	Type of amplification
Child 1	Female	2.1	2.1	Normal hearing	N/A^b^
Child 2	Female	2.3	2.3	Normal hearing	N/A
Child 3	Male	2.5	2.5	Normal hearing	N/A
Child 4	Male	2.5	2.5	Normal hearing	N/A
Child 5	Male	2.5	2.5	Normal hearing	N/A
Child 6	Male	1.9	1.9	Normal hearing	N/A
Child 7	Female	2	2	Normal hearing	N/A
Child 8	Male	2.8	2.8	Normal hearing	N/A
Child 9	Male	2.9	2.9	Normal hearing	N/A
Child 10	Female	1.8	1.8	Normal hearing	N/A
Child 11	Male	3.7	1.5	Bilateral: Moderately severe hearing loss	Bilateral: Hearing aid user
Child 12	Female	4	1.8	Bilateral: Severe to profound hearing loss	Right: Cochlear implant
Child 13	Male	3.8	1.7	Bilateral: Severe hearing loss	Right: Cochlear implant; left: Hearing aid
Child 14	Female	4.9	2.2	Bilateral: Severe to profound hearing loss	Left: Cochlear implant
Child 15	Female	4.2	1.7	Bilateral: Moderately severe hearing loss	Bilateral: Hearing aid

^a^SSAT: speech spectrum awareness task.

^b^N/A: not applicable.

##### SRT-Based Screening

Twenty children (15 children with hearing impairment and 5 children with normal hearing between the ages of 3 to 9 years with a mean age of 3.7 (SD 0.44) years for children with normal hearing and 3.8 (SD 0.94) years of hearing age for children with hearing impairment (Table 7) underwent the speech recognition task using the spondee words.

**Figure 3:**
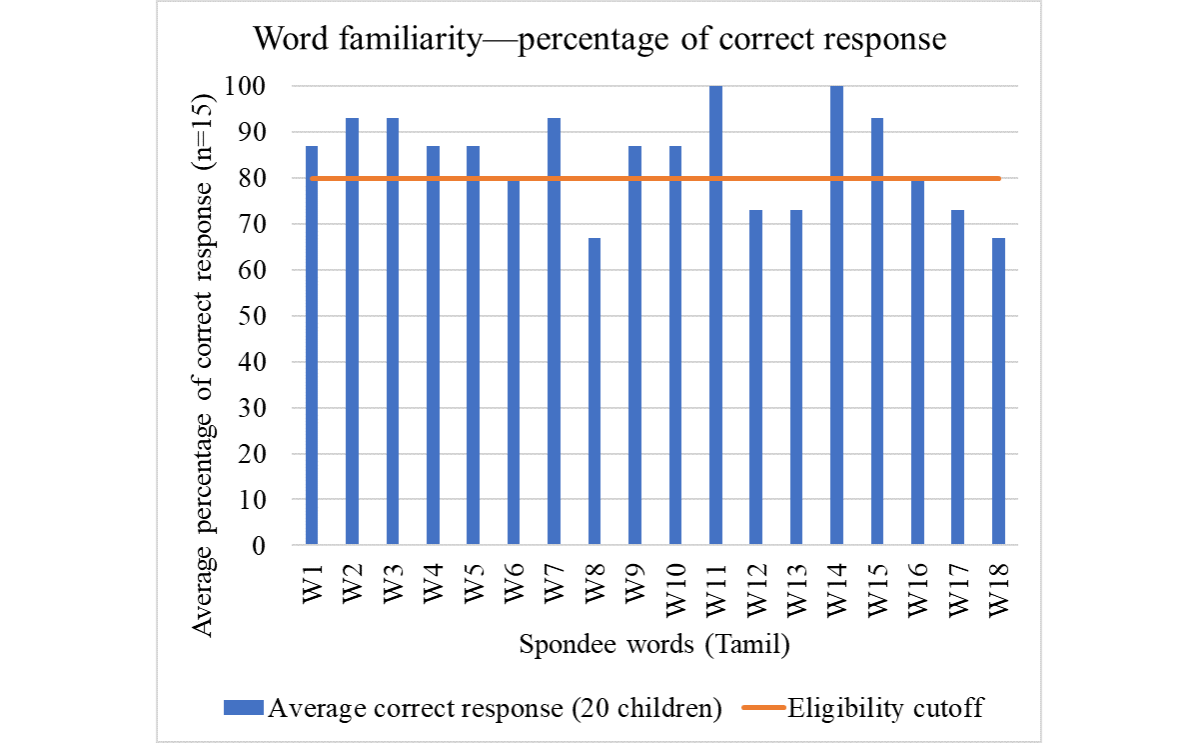
Average percentage of correct responses by children for each word.

Twelve of the 18 words could be identified by 85% of the normal-hearing children. Therefore, these words were included as 2 lists of 6 words each (Figure 3).

**Figure 4:**
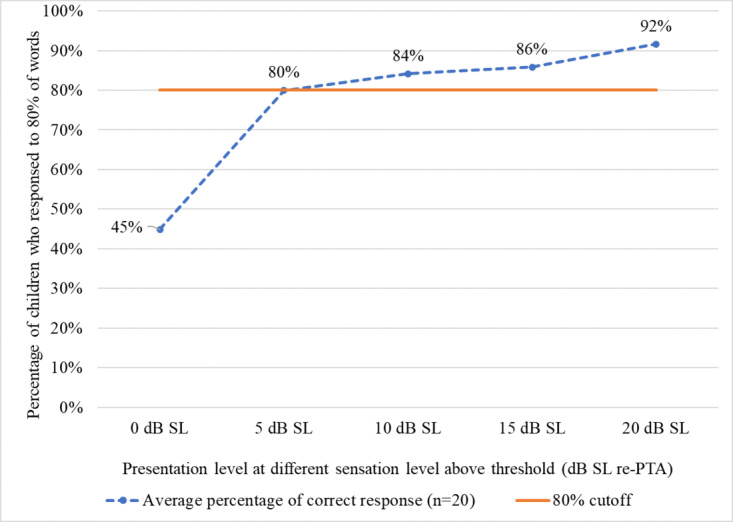
Comparison of percentage correct responses versus speech recognition threshold of 20 children. PTA: pure tone threshold.

Thresholds for warble tone were compared with the speech recognition threshold to find the suitable presentation level for the spondee words for 80% psychometric function, 5 dB SL (re PTA) was found to be sufficient (Figure 4).

**Table 7:** Participants characteristics of children involved in SRT^a^ stimuli validation.

Participants	Sex	Age (years)	Hearing age (years)	Diagnosis	Type of amplification
Child 1	Female	3.3	3.3	Normal hearing	N/A^b^
Child 2	Female	4.3	4.3	Normal hearing	N/A
Child 3	Male	4.2	4.2	Normal hearing	N/A
Child 4	Male	3.5	3.5	Normal hearing	N/A
Child 5	Male	3.6	3.6	Normal hearing	N/A
Child 6	Female	5.8	3	Bilateral: Severe to profound hearing loss	Right: Cochlear implant
Child 7	Male	4.1	3	Bilateral: Severe to profound hearing loss	Left: Cochlear implant
Child 8	Female	4.5	3.1	Bilateral: Moderate to moderately severe hearing loss	Bilateral: Hearing aid user
Child 9	Female	5	3.5	Bilateral: Profound hearing loss	Left: Cochlear implant
Child 10	Female	4.2	3	Right: Moderately severe hearing loss; left: Severe hearing loss	Left: Cochlear implant
Child 11	Female	8.5	5.2	Bilateral: Moderately severe hearing loss	Bilateral: Hearing aid user
Child 12	Male	7.1	4.5	Bilateral: Severe to profound hearing loss	Right: Cochlear implant
Child 13	Male	8	5.5	Bilateral: Moderately severe hearing loss	Right: Cochlear implant; left: Hearing aid
Child 14	Male	6.5	4.1	Left: Moderately severe hearing loss; right: Severe hearing loss	Bilateral: Hearing aid user
Child 15	Female	5	3.5	Bilateral: Moderately severe hearing loss	Bilateral: Hearing aid user
Child 16	Female	9	5	Bilateral: Severe to profound hearing loss	Left: Cochlear implant
Child 17	Female	7.5	3.2	Bilateral: Moderately severe hearing loss	Right: Cochlear implant
Child 18	Male	4.6	3	Bilateral: Severe hearing loss	Left: Cochlear implant
Child 19	Male	5.2	3.1	Bilateral: Severe to profound hearing loss	Right: Cochlear implant
Child 20	Male	4	3	Bilateral Severe to profound hearing loss	Left: Cochlear implant

^a^SRT: speech recognition task.

^b^N/A: not applicable.

### Accuracy of SRESHT Screening Modules: Beta Validation

A total of 55 children (31 children with normal hearing and 24 children with hearing impairment underwent hearing screening using the SRESHT screener device. The chronological age range for children with normal hearing participants was 2 days to 6 years with a mean age of 3.2 (SD: 1.94) years, whereas the chronological age range for children with hearing impairment participants was 1 year to 9 years with a mean age of 4.9 (SD 1.42) years.

Of the 24 children with hearing impairment, 14 were unilateral cochlear implantees, 8 were bilateral hearing aid users, and 2 used bimodal hearing devices. The diagnostic reports of all 24 children with hearing impairment were reviewed to verify their unaided and most recent aided thresholds. Table 8 shows the agreement results.

Therefore, the SRESHT screener modules with new stimuli were as good as the standard audiometer in identifying moderately severe and higher degrees of hearing loss of 60 dB HL or higher.

**Table 8:** Agreement in findings between SRESHT screener and gold standard audiometric screening.

Test frequencies	κ value	95% CI	*P* value	Agreement
500 Hz	1.00	1.00-1.00	0.001	Perfect agreement
1000 Hz	0.87	0.75-1.00	0.001	Very good agreement
2000 Hz	0.72	0.47-0.97	0.005	Good agreement
4000 Hz	0.85	0.75-1.00	0.001	Very good agreement
Overall	0.82	0.66-0.97	0.001	Very good agreement

## Discussion

### Principal Findings

This study focused on the development and beta validation of an affordable subjective hearing screener (SRESHT) for identifying hearing losses greater than 60 dB HL in children younger than 6 years of age. The SRESHT hearing screener was developed in a single-board computer with calibrated off-the-shelf transducers. For children between 0 and 1 year of age, the BOA module was developed with noisemakers as suitable stimuli. For children between 1 and 3 years of age, the SSAT module with animal sounds was found as a suitable stimulus. For children between 3 and 6 years of age, the SRT module with 12 Tamil spondee words was found suitable. On beta validation of the SRESHT screener with age-appropriate modules among children between 0 to 6 years of age, it was found that the SRESHT screener was suitable to identify hearing losses of moderately severe and higher degrees.

The SRESHT screener is an affordable alternative with age-appropriate subjective hearing screening modules for children from birth to 6 years of age. The cost estimate is expected to be one-tenth of an objective hearing screener. Among the available smartphone-based hearing apps, only a few are designed specifically for children, but all are applicable only for children older than 4 years of age [18,21,24,43]. So far, to our best knowledge, the clinical population of children with hearing impairment has not been included in studies that describe mHealth hearing screeners.

In high-income countries, objective screening methods such as otoacoustic emissions and auditory brainstem response are routinely used as part of universal hearing screening as they are more accurate [26]. In LMICs, while there are efforts to use objective screeners by private hospitals and research teams, nationwide programs are negligible [2,44,45]. In a pilot government-initiated newborn hearing screening program in a southern state in India, objective screeners were initially procured, however, its long-term viability was called into question due to the high cost of screening equipment and frequent repair and consumables costs [46-48].

The semiautomated SRESHT screener is currently designed to screen “moderately severe” or higher degrees of hearing loss [49]. As a first step, this higher level of screening was chosen as part of a feasibility study to make a valid tool available to screen for 60 dB HL in the better ear (eligibility for publicly funded welfare schemes) within the public health system of a southern state in India. Not only in India, but in several LMICs (Bangladesh, Pakistan, and Kenya), a criterion of 50 dB HL or higher is used to provide welfare schemes, aid, and appliances with public funds. As a result, affordable tools that support this level of screening are useful in identifying this group of children who would otherwise miss out on welfare measures.

In the past, subjective screening using distraction and whisper tests in the United Kingdom [50,51], informal clap screening in Africa [52], noisemaker-based screening in India [53], and questionnaire-based screening in Thailand [5] and Kenya [7] have been used to support mass screening in the community. Some of the drawbacks of these screening methods are the lack of calibrated stimuli, the requirement of 2 personnel to administer the test, lack of standardization of intensity, frequency, distance, and test-retest reliability [11,26-28]. The SRESHT screening with age-appropriate calibrated stimuli reduced the extent of subjectivity. For the youngest babies, noisemakers are known to be robust [31,36,54] and this was reconfirmed from our beta validation. Stimuli such as animal sounds are known to be appealing to younger children for hearing testing [55], therefore, animal sounds were mapped to speech spectrums to develop the speech spectrum awareness test. These 2 modules are language independent and therefore can be used in several resource-limited settings across the world to support community-based screening efforts.

For the youngest age group (0 to 1 year), noisemakers are known to be robust [31,54], and this was reconfirmed from the results of phase 1 of the study and used for the BOA screening module. In this study, BOA screening was conducted with noisemakers for infants under the age of 1 year. Visual reinforcement audiometry screening or distraction test is recommended for children aged 6 months to 1 year in a few studies [54] and guidelines [11,56], however, since the SRESHT screener is conceptualized for use by community workers and therefore the level of complexity and training required was an important consideration to choose BOA over visual reinforcement audiometry.

The initial version of the screening test for the age group of 1 year to 3 years consisted of a visual reinforcement task using warble tones. A simple light-emitting toy with a manual switch control was also developed for visual reinforcement. However, during field testing, the grassroots-level workers could not perform the screening task efficiently and provided feedback on practical challenges. This led to the modification of the module to a speech spectrum awareness task. The task was also tried in commercial grade insert earphones. However, insert earphones were not found suitable as children between 1 and 3 years of age were not comfortable to wear them.

For older children, the SRESHT screener uses picturable age-appropriate spondee words in the local language. While many apps have used pure tones [18,57]; warble tones [22,43], and very few exist with speech stimuli [21,24]. Picturable spondee words of different languages can be added to make the screener available elsewhere.

Affordable screeners are much needed to support nationwide early intervention programs in LMICs for long-term viability. The cost of screening a child for hearing loss using objective screening methods is estimated to be between US $17 and US $26 across high-income countries and LMICs like Brazil [58], India [59], the United States [60], and China, [61,62]. This cost can be lowered when device costs are less. Hearing screening apps for adults and older children range in price from US $600 to US $2000 [63], and some require standard audiometric headphones [18,22,24]. Commercial headphones with precise calibrations, on the other hand, can produce consistent output [16,17,21]. To keep costs low, the SRESHT screener was developed using off-the-shelf transducers that produce standard output with RETSPL derivation thereby reducing the cost to one-tenth the price of objective screening tools.

In community-based, school-based or mass screening programs, in-built noise monitoring is crucial due to the lack of sound-treated booths. The SRESHT screener performs noise monitoring prior to the commencement of each of the screening tests to limit the contamination of results by high ambient noise levels. Such features exist in some of the app-based screeners already [23,43,64].

The SRESHT screener is currently limited to permanent hearing losses of moderately severe and higher degrees in children under 6 years of age. Hence, further study is required to extend the scope of the screener to all types and degrees of hearing loss. While the scope of the current study is limited to the development of the screening modules with suitable beta validation, the full validation of the hearing screener with these finalized parameters has been undertaken and will be reported as a separate paper.

### Future Directions

Full validation against the gold standard objective test is undertaken for this device and the sensitivity, specificity, and positive and negative predictive values will be reported in a separate publication. A project is also being undertaken to lower the screening intensity levels to screen for lower degrees of hearing loss.

## Abbreviations

BOA: behavioral observation audiometry
HL: hearing loss
LMIC: low- and middle-income country
mHealth: mobile health
PTA: pure tone threshold
RETSPL: reference equivalent threshold sound pressure level
SLM: sound level meter
SRT: speech recognition task
SSAT: speech spectrum awareness task

## Data Availability

The datasets generated during and analyzed during this study are available from the corresponding author on reasonable request.
